# Editorial Perspective: How can we develop effective and timely interventions for young people with chronic loneliness?

**DOI:** 10.1111/jcpp.14097

**Published:** 2024-12-21

**Authors:** Tom Cawthorne, Pamela Qualter, Sophie Bennett, Anton Käll, Gerhard Andersson, Roz Shafran

**Affiliations:** ^1^ South London and Maudsley NHS Foundation Trust London UK; ^2^ Manchester Institute of Education, University of Manchester Manchester UK; ^3^ Kings College London London UK; ^4^ Department of Behavioural Sciences and Learning Linköping University Linköping Sweden; ^5^ Department of Biomedical and Clinical Sciences Linköping University Linköping Sweden; ^6^ Department of Clinical Neuroscience Karolinska Institute Stockholm Sweden; ^7^ UCL Great Ormond Street Institute of Child Health London UK

**Keywords:** Loneliness, intervention, prevention, mental health, therapy

## Abstract

Loneliness is an adaptive experience evolved to create motivation to engage in social relationships. However, for some young people, loneliness can become chronic which can have serious negative health consequences. Despite this, there is a relative lack of evidence for interventions. In this editorial perspective, we highlight four main barriers to the timely development and dissemination of evidence‐based support for young people experiencing loneliness. We hypothesise that these challenges could be mitigated by (a) routinely assessing loneliness as part of routine outcome measures (ROMs), (b) utilising modular interventions incorporating intrapersonal, interpersonal and social strategies alongside system‐level support and policy changes, (c) evaluating interventions through SCEDs prior to RCTs and (d) delivering interventions flexibly (e.g. via the internet or within non‐clinical settings).

## Introduction

Loneliness is a typical and expected experience among young people exacerbated by the developmental challenges of adolescents such as the transition to adulthood, social pressures and identity formation. For many, loneliness serves an adaptive function because it provides motivation to engage in social relationships, but for approximately 20% of young people, it can become a chronic problem (Qualter et al., 2021). Such chronic loneliness is predictive of multiple physical and mental health difficulties (Hards et al., 2022; Qualter et al., 2021). However, despite the seriousness of the concern, evidence‐based interventions for loneliness in young people are not routinely available and recommendations by healthcare bodies are a long way off. In this editorial perspective, we highlight what we perceive as the main challenges that must be overcome to achieve that goal.

### Challenge 1: Young people are not currently routinely screened for loneliness

Chronic loneliness is a particular problem for children and young people with pre‐existing mental health problems (Hards et al., 2022), but clinical services do not routinely assess it. This has contributed to our lack of understanding regarding how those with problematic loneliness should be supported. Data on loneliness is not routinely collected in child and adolescent mental health services (CAMHS); this is a missed opportunity because services can use routine outcome measures (ROMs) at multiple time points to evaluate intervention outcomes. Including a loneliness measure within the ROMs would allow for better characterisation of loneliness within the clinical population; we could then identify the prevalence of chronic loneliness among youth accessing mental health services, and whether there are differences in loneliness prevalence and aetiology across different clinical subgroups. Similarly, screening for loneliness within schools may also allow for the earlier identification of young people with transient loneliness likely to later develop chronic problems. Understanding the extent to which loneliness is a problem within a given population (e.g. a school) could then lead to the provision of preventative and earlier support outside of a clinical context, where it may not be feasible or beneficial to provide interventions within an evidence‐based medicine paradigm. This is of particular importance because it is not helpful to categorise loneliness as a clinical condition that can only be ‘treated’ within healthcare services. Instead, loneliness is a multifaceted issue that must be addressed across multiple levels, including national and local policies and through social and community initiatives that work alongside mental health services.

Measuring loneliness within ROMs also allows for the assessment of whether loneliness is predictive of differential intervention outcomes when young people receive interventions for their primary mental health problem. If loneliness is found to predict worse outcomes, providing this population with an intervention specifically targeting loneliness may lead to an improved prognosis; alternatively, if loneliness is found to predict differential relapse rates, young people with elevated loneliness would require intervention for loneliness following treatment for their primary mental health problem.

Routinely screening young people for loneliness would be possible. As there are several validated measures of loneliness in children and young people (see Cole, Bond, Qualter, & Maes, 2021 for review). One of these measures, the three‐item loneliness scale (ONS, 2018), is also very brief, reducing the burden on resources for administration and scoring; it is also acceptable and feasible for use as a weekly outcome measure for 11–18‐year‐olds (Cawthorne, Käll, Bennett, Baker, Andersson, et al., 2023).

### Challenge 2: The population of young people at an elevated risk of loneliness is highly heterogeneous

The population of young people presenting with an elevated risk of problematic loneliness is highly heterogeneous and includes those with chronic health problems, mental health difficulties and those on the autism spectrum (Hards et al., 2022). Due to this significant heterogeneity, it is likely that a ‘one‐size‐fits‐all’ approach will not be effective for addressing chronic loneliness across this diverse population.

A recent critical interpretive synthesis identified three types of effective interventions, based on ‘mechanisms of action’ (Pearce et al., 2021): intrapersonal strategies (e.g. psychological therapy focussed on changing thinking and behaviour), interpersonal strategies (e.g. social skills training) and social strategies (e.g. enhancing social support and promoting opportunities for social contact). Such differential interventions may prove effective for different groups of youth presenting with loneliness. However, such an approach would be costly: each intervention would need to be developed before evaluation using large‐scale clinical trials sufficiently powered to determine whether the intervention is effective and for whom.

An alternative and more cost‐effective approach are modular interventions. Such an intervention provides different modules that are applied flexibly to different groups of people dependent on the factors that are maintaining their loneliness. The modular approach also provides a framework for addressing multiple maintenance factors not achieved by a single non‐modular intervention. For instance, if you were supporting a young person whose loneliness was maintained by social skills difficulties associated with autism and co‐occurring social anxiety, a modular approach would provide an evidence‐based framework for providing social skills training, followed by a CBT intervention addressing their anxious cognitions. If the client then developed friendships, but found their new friends liked to spend time in a busy shopping centre, which the client felt unable to manage due to sensory overarousal, a problem‐solving module could then be used to identify possible solutions (e.g. wearing headphones or asking if their friends wanted to go to the park instead). Additional components focussed on environmental adaptations and parent‐led support could also be included, maximising the inclusivity of the intervention and ensuring appropriateness for young people of different ages, developmental levels and neurodiversity. As with any intervention, a modular approach would need to be situated in a wider understanding of the difficulties. Additional support should be offered throughout the system as needed, for example, children and young people who are identified as being neurodivergent may benefit from adaptations to the school environment.

We recently developed CBT for Chronic Loneliness in Young People (Cawthorne, Käll, Bennett, Baker, Andersson, et al., 2023) based on a modular framework. The intervention included modules spanning each of the hypothesised ‘mechanisms of action’ (Pearce et al., 2021). We evaluated this intervention using a single‐case experimental design (SCED), finding large effect sizes for reducing both loneliness and co‐occurring mental health difficulties in a group of 11–18‐year‐olds. Internet‐delivered modular interventions have also been shown to be effective in reducing loneliness in adults in several RCTs (Käll et al., 2021), but both interventions lack modules focussed on social prescribing, which has been identified as another important intervention mechanism (Pearce et al., 2021). Future research should seek to build upon these modular protocols, incorporating additional modules on social prescribing and environmental adaptations that work alongside wider systemic interventions and policy changes.

The modular design is not without its challenges. For example, it is rarely possible to identify which modules are individually effective and it is difficult to determine the precise module(s) that should be deployed even when maintaining mechanisms are identified. However, these are similar challenges in non‐modular approaches because clear evidence on exactly which specific components of most evidence‐based interventions are effective is currently lacking. The person‐centred nature of psychological support also results in variability in the intervention experience of service users even within highly protocolised interventions.

### Challenge 3: The length of time taken for research evidence to reach clinical practice

We argue SCEDs provide a high‐quality, low‐resource alternative to RCTs: they overcome the 17 years it currently takes for research evidence to reach clinical practice (Cawthorne, Käll, Bennett, Baker, Cheung, et al., 2023). SCEDs also provide a methodologically rigorous alternative to other group designs (e.g. case series), allowing for causal inferences to be made (Kazdin, 2021).

The SCED approach could be used to accelerate the identification of effective interventions for loneliness in young people in several ways. Firstly, SCEDs allow for greater focus on practice‐based evidence by providing a framework for high‐quality evaluation of interventions currently being conducted within services. Due to the smaller scale and lower resource requirements of a SCED in comparison to RCTs, it would be relatively straightforward to conduct these trials as part of routine clinical practice. For instance, it may not be necessary to apply for funding or recruit research teams, which all contribute to delays in the development of evidence‐based interventions.

The flexible design of SCEDs means that they can be used to evaluate a variety of intervention procedures to reduce loneliness across both clinical and non‐clinical settings (e.g. schools). This flexibility in intervention delivery is paramount as whilst for some young people an effective intervention for loneliness may be psychological therapy, for others it may be the opportunity to learn a new skill, or to join a social group (Pearce et al., 2021). The adaptability of the SCED design would also allow for the evaluation of wider system‐level interventions (e.g. school‐based initiatives) with the effect of the intervention specifically on young people experiencing problematic chronic loneliness being evaluated, even where support is made universally available.

The ability to deliver and evaluate interventions across these different settings would allow us to more effectively support young people with transient difficulties before they develop chronic problems, as well as young people who meet the threshold for clinical services. More complex SCED designs would also allow for comparisons between intervention‐types, or for the examination of how different interventions (or intervention modules) work when conducted in sequence or in different combinations.

SCEDs also provide idiographic detail and richness alongside methodological rigour; they generate hypotheses regarding what interventions work for different groups of young people (Kazdin, 2021). Following SCED evaluation, these interventions can then be more robustly evaluated through RCTs and those hypothesise tested. Due to the high resource burden of RCTs, it is also not feasible to evaluate all interventions using this methodology; SCEDs provide an alternative high‐quality design for the generation of evidence of sufficient quality to inform intervention recommendations when RCTs are unsuitable.

### Challenge 4: The lack of access to timely evidence‐based interventions

Mental health problems in children and adolescents are now recognised as a global concern. However, timely access to evidence‐based interventions is impacted by long waitlists, staff shortages and concerns regarding clinician fidelity to evidence‐based therapies. These difficulties have since been exacerbated by the pandemic and subsequent global economic problems. This means that should effective evidence‐based intervention(s) for loneliness for youth be made available, there are barriers to access for young people in real‐world clinical services. We therefore need to identify alternative ways for young people to access such interventions to reduce their loneliness outside of the traditional therapeutic paradigm.

An internet‐delivered CBT intervention for adults has been shown to be effective at reducing loneliness in several RCTs (Käll et al., 2021). Several intervention modules from this intervention have been adapted and incorporated into a recent face‐to‐face intervention, proving effective for young people (Cawthorne, Käll, Bennett, Baker, Andersson, et al., 2023). Thus, internet‐delivered interventions for loneliness are helpful for children, adolescents and adults reporting chronic loneliness. They also reduce barriers to access: there is less clinician time and training, they can be delivered to larger numbers of children and adolescents simultaneously and may be more acceptable to young people who feel unable or are unwilling to access support face‐to‐face.

A second priority should be the development and evaluation of loneliness interventions outside of the clinical context (Pearce et al., 2021). Delivering loneliness interventions outside of clinical services may increase access and acceptability because they are less dependent on scarce clinical resources and would be available for those who do not meet the threshold for clinical services. By providing loneliness interventions within non‐clinical environments, for example, schools or youth groups, loneliness stigma is also reduced, making it more acceptable for young people to report loneliness and request support.

## Summary

Loneliness is a common problem in young people associated with a range of physical and mental health problems and increased mortality. However, there are significant barriers to the timely development and dissemination of effective interventions despite the urgency of this concern. In this editorial perspective, we highlighted what we perceive as the four main barriers to achieving this goal. We hypothesised that those challenges are minimised or overcome through a range of strategies, including (a) routinely assessing loneliness as part of routine outcome measures (ROMs), (b) utilising modular interventions incorporating intrapersonal, interpersonal and social strategies alongside system‐level support and policy changes, (c) evaluating interventions through SCEDs prior to RCTs and (d) delivering interventions flexibly via the internet or within non‐clinical settings.

## Data Availability

Data sharing is not applicable to this article as no datasets were generated or analysed during the current study.
